# Gastrocnemius medialis muscle architecture and its relationship with countermovement jump performance differ between male academy soccer players and control participants

**DOI:** 10.1007/s00421-026-06311-9

**Published:** 2026-06-29

**Authors:** David C. Robshaw, Conall F. Murtagh, Barry Drust, Robert M. Erskine

**Affiliations:** 1 School of Sport and Exercise Sciences, Liverpool John Moores University, Liverpool, L3 3AF UK; 2 School of Sport, Exercise and Rehabilitation Sciences, University of Birmingham, Birmingham, UK; 3 Institute of Sport, Exercise and Health, University College London, London, UK

**Keywords:** Plantar flexors, Football, Countermovement jump (CMJ), Drop jump, Muscle architecture

## Abstract

The aims of this study were to investigate gastrocnemius medialis (GM) muscle properties in male academy soccer players (ASP) and age- and sex-matched control participants (CON); and to explore the relationships between GM characteristics and jump performance. Thirty-four participants (ASP, *n* = 22, age 18.8 ± 1.4 years, height 1.82 ± 0.08 m, mass 75.1 ± 5.9 kg; and CON, *n* = 12, 22.2 ± 2.9 years, 1.75 ± 0.05 m, 71.6 ± 7.4 kg) completed the following assessments: ultrasound measurements of GM anatomical cross-sectional area (ACSA), volume, muscle thickness (MT), fascicle pennation angle (*θ*_p_) and fascicle length (*L*_f_); isokinetic dynamometry measurements of isometric plantar flexion and dorsiflexion maximum voluntary torque; and unilateral and bilateral, vertical and horizontal, countermovement jumps (CMJ), and bilateral drop jumps on a force platform. *θ*_p_ (17.4° ± 2.5° vs. 14.3° ± 1.2°, *P* < 0.001); unilateral horizontal CMJ peak power (30.14 ± 3.53 vs. 23.18 ± 3.72 W kg^− 1^); and projectile range during unilateral (104 ± 16 vs. 89 ± 12 cm, *P* = 0.006) and bilateral (140 ± 14 vs. 129 ± 14 cm, *P* = 0.041) horizontal CMJ were greater in ASP vs. CON. In ASP alone, *L*_f_ correlated *inversely* with vertical CMJ performance but *positively* with horizontal CMJ performance (*R*^2^ ≥ 0.200, *P* ≤ 0.042). Conversely, *θ*_p_ correlated *positively* with vertical CMJ performance but *inversely* with horizontal CMJ performance (*R*^2^ ≥ 0.194, *P* ≤ 0.044). In CON only, ACSA, MT, volume and *L*_f_ all correlated *inversely* with vertical CMJ performance (*R*^2^ ≥ 0.366, *P* ≤ 0.037). The opposing *θ*_p_ and *L*_f_ correlations with vertical and horizontal CMJ jump performance in ASP suggest GM architecture influences CMJ performance in a direction-specific manner in this population, while the different correlation patterns between ASP and CON suggest that GM architecture contributes to CMJ performance differently in these two populations.

## Introduction

Physical profiling of elite athletes is often performed to identify influential characteristics of key performance indicators (KPI) of the sport. Acceleration and maximal sprinting are well known KPIs in competitive soccer (Emmonds et al. 2016; Faude et al. 2012), and are underpinned by power and reactive strength qualities (Colyer et al. 2018; Douglas et al. 2020; Morin et al. 2012). Related to this, countermovement jumps (CMJ) and drop jumps have been implemented in physical profiling to investigate the determinants of power (Murtagh et al. 2017, 2018a, b; Murtagh et al. 2018a, b; Robshaw et al. 2025) and reactive strength performance (Douglas et al. 2020; Maloney et al. 2018).

Previous studies have sought to identify the neuromuscular characteristics that underpin performance in jumping tasks. One of the most consistent findings has been the relationship between muscle size (thickness and physiological cross-sectional area) of the vastus lateralis (VL) muscle (Mangine et al. 2014; Secomb et al. 2015a, b; Murtagh et al. 2018a, b) and the lateral gastrocnemius (LG) head (Earp et al. 2010; Secomb et al. 2015a, b) with jump variables (e.g. height, power, peak force) during squat jumps, CMJs, and drop jumps. Further, positive relationships have been observed between VL and LG fascicle pennation angle [*θ*_p_, i.e. the angle at which the muscle fascicles insert into the lower aponeurosis, thought to be determined by muscle fibre cross-sectional area (Aagaard et al. 2001; Degens et al. 2009)], and jump height, mean power, peak power and peak velocity in vertical jumps (Alegre et al. 2005; Earp et al. 2010; Secomb et al. 2015a, b; Murtagh et al. 2018a, b).

However, inconsistent reports have been published following training interventions, where increases in muscle size have occurred without changes to jump height (Fouré et al. 2010), whilst others have shown jump height to improve in the absence of changes in muscle size (Blazevich et al. 2003). Rather than contradicting previous reports, these findings could highlight the importance of determining peak power during a jump rather than simply measuring the height or distance attained. This could be of particular importance given that peak power can help differentiate athletic status when jump height cannot (Murtagh et al. 2017). Similarly, vertical stiffness (i.e. the peak centre of mass displacement relative to the peak ground reaction force, GRF) and reactive strength index (RSI, i.e. the ratio of flight time to contact time) rather than jump height could also be considered KPIs during a drop jump (Douglas et al. 2020). However, limited research exists exploring the potential influence muscle architecture has on these parameters, favouring instead to report relationships only with jump height (Earp et al. 2010, 2011).

Furthermore, there is a distinct lack of research identifying the important characteristics of the gastrocnemius medialis (GM) head for jump performance (Dobbs et al. 2015; Pentidis et al. 2020). Findings from studies investigating the VL cannot be generalised to all muscles due to the different behaviour of fascicles and tendinous structures during jumping tasks (Ishikawa et al. 2005; Sousa et al. 2007). Therefore, given the importance of the plantar flexors in jumping and running based tasks (Bobbert et al. 1986; Dorn et al. 2012; Hamner and Delp 2013; Hamner et al. 2010; Kipp et al. 2018; Maloney et al. 2016; Schache et al. 2019), which are important for soccer performance, further research is necessary to determine if GM characteristics differ between soccer players and control participants, and to understand if GM properties can influence performance in jumping actions pertinent to soccer. Such information could be used by coaches to identify talented academy soccer players and monitor their development over time. Furthermore, it may inform the design of more targeted training interventions in academy soccer players, e.g. specific plantar flexor resistance training to help improve training and match performance.

As previously mentioned, the physical characteristics expressed during a jump should be the focus of assessment rather than the distance obtained, however, the reliability of power calculated during CMJs has been shown to differ according to the direction and specific phase of the jump (Meylan et al. 2010; Robshaw et al. 2025). Similarly, limited data exist that compare vertical stiffness and RSI during a drop jump, despite their strong associations with sprinting performance (Douglas et al. 2020; Kipp et al. 2018; Maloney et al. 2018). In addition, whilst GM morphology has been investigated in a variety of populations, inconsistent reliability has been reported for muscle thickness (MT), fascicle length (*L*_f_) and *θ*_p_ (Dudley-Javoroski et al. 2010; Legerlotz et al. 2010; McMahon et al. 2016; Mohagheghi et al. 2007; Raj et al. 2012). This inconsistency can make generalising findings difficult.

The aims of this study were therefore to (i) investigate the test-retest reproducibility of GM muscle property assessments; (ii) investigate plantar flexor strength and GM muscle morphology and architecture in adult male (to control for sex-related physiological differences) academy soccer players (ASP) and recreationally active age- and sex-matched control participants (CON), partaking in recreational soccer no more than twice per week (i.e. less than ASP) and not playing for a professional soccer club academy; and (iii) explore the relationship between GM muscle characteristics and performance outcomes in a series of jumping tasks. It was hypothesised that muscle size and *θ*_p_ would be larger in ASP than CON, and that these muscle variables would correlate positively with peak power generated during CMJs, whilst plantar flexor force would correlate with vertical stiffness. By focussing on male ASP and CON, this study will address an important knowledge gap and provide age-, sex- and sport-specific evidence for researchers and practitioners working with male ASP.

## Method

### Participants

Thirty-four healthy young men, comprising 22 under 23 (U23) and U18 academy soccer players (ASP; age = 18.8 ± 1.4 years, height = 1.82 ± 0.08 m, mass = 75.1 ± 5.9 kg; mean ± SD) and 12 physically active CON (age = 22.2 ± 2.9 years, height = 1.75 ± 0.05 m, mass = 71.6 ± 7.4 kg), who took part in recreational soccer no more than twice per week and had not played for a professional soccer club academy, volunteered to take part in this study. Participants were classified as ASP if they competed at U18 or U23 level soccer in an English Premier League Category 1 soccer academy and were free from any lower limb injury. CON comprised recreationally active men, who were free from any lower limb musculoskeletal injury within the three months prior to the start of the study. All participants provided written informed consent to take part in this study. Ethical approval was provided by Liverpool John Moores University Research Ethics Committee (approval number: 17/SPS/030) and the study complied with the Declaration of Helsinki.

### Experimental design

Participants attended the laboratory on two separate occasions, each one at least 48 h apart (maximum 7 days), to complete a familiarisation session (60 min) and data collection session (120 min). The latter session was at least 48 h after any strenuous exercise. During the first session, participants were familiarised with the muscle function assessments, which comprised at least three plantar flexion (PF) and dorsiflexion (DF) maximal voluntary isometric contractions (MVCs) of the dominant leg (their preferred kicking leg) on an isokinetic dynamometer (IKD). Following this, participants were familiarised with a series of bilateral and unilateral (dominant leg only) vertical and horizontal CMJs, as well as bilateral vertical drop jumps. During the second session, participant standing height and body mass were recorded, along with measurements of GM muscle architecture (*L*_f_ and *θ*_p_) and size (MT and anatomical cross-sectional area, ACSA) using ultrasound. Muscle volume (*V*_m_) and physiological cross-sectional area (PSCA) of the GM were assessed in CON. Participants were then positioned on the IKD and performed a series of PF and DF isometric MVCs. Participants subsequently performed a standardised warm-up protocol, comprising several dynamic movements and stretches, before completing two practice trials of each jump type. Participants then performed three trials of each jump, with their arms akimbo to minimise inter-individual differences in technique influencing performance. All jumps were performed on a force plate to record GRF.

A sub-sample of 10 CON participants attended the laboratory for a third time (2–7 days following the second visit) and were used to determine between-session reproducibility for all independent variables. Reproducibility was assessed using a two-way random effects model (absolute agreement) intraclass correlation coefficient (ICC), as well as ratio limits of agreement (Nevill and Atkinson 1997). Absolute reliability for all independent variables was reported as typical error (TE), while relative reliability was calculated using coefficient of variation (CV, %) (Hopkins 2000). TE was calculated using the following equation:

$$Standard Deviation of Differences / \left( {\surd 2} \right)$$

CV was determined using the following equation:

$$100 * \left( {\surd \left( {Sum of squared differences /\left( {2* sample size} \right)} \right)/\left( {Average of measurement 1 and 2} \right)} \right)$$

Due to loss of image quality in a small number of participants, the data from 10 CON participants were used in the analysis of muscle volume and PCSA.

### Muscle size and architecture

**Fig. 1:**
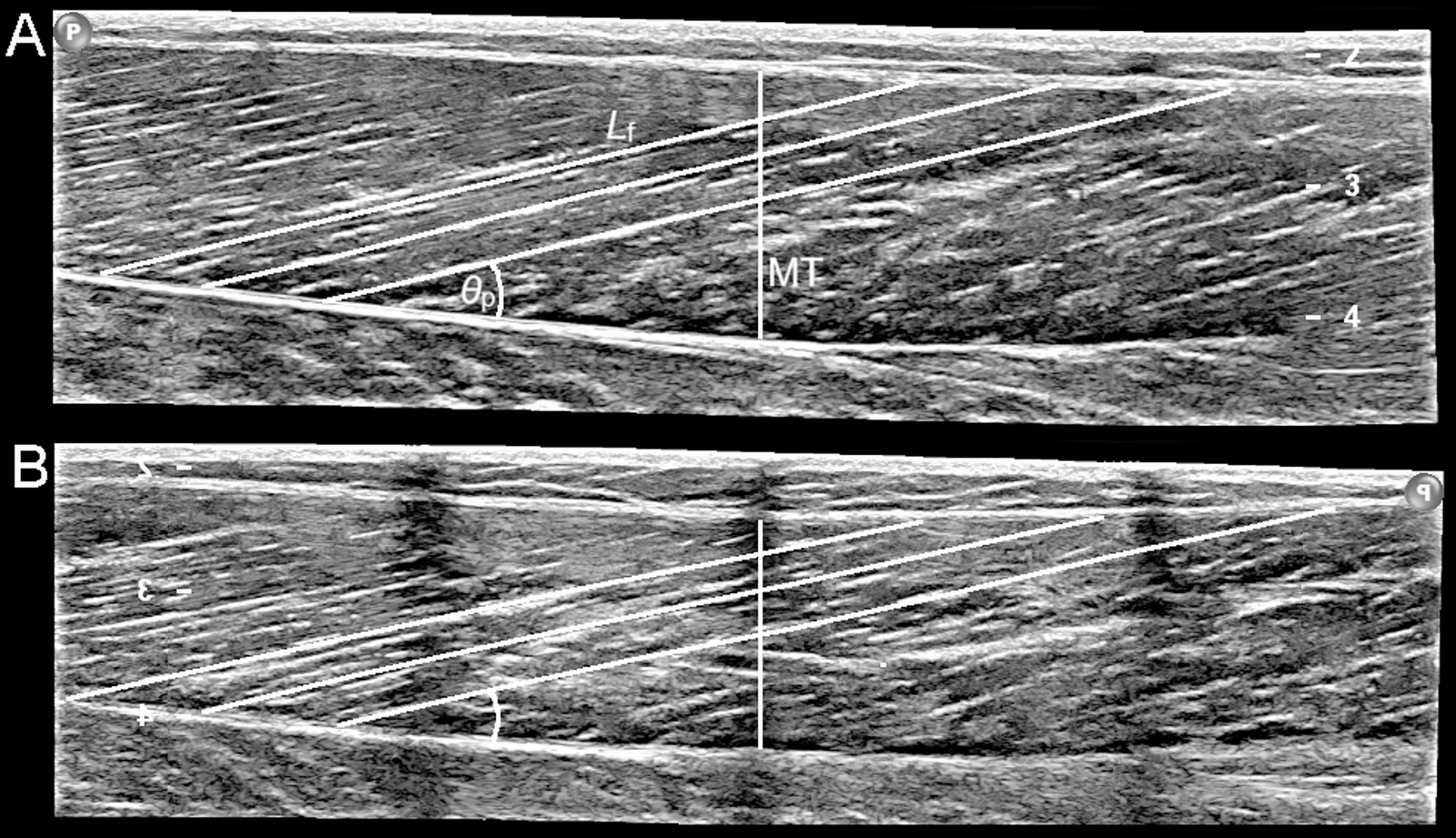
Representative images of the gastrocnemius medialis (GM) muscle architecture of an academy soccer player (**A**) and a control participant (**B**). Highlighted in each image is the measurement of muscle thickness (MT) at 50% GM length, fascicle length (*L*_f_) and fascicle pennation angle (*θ*_p_)

**Fig. 2:**
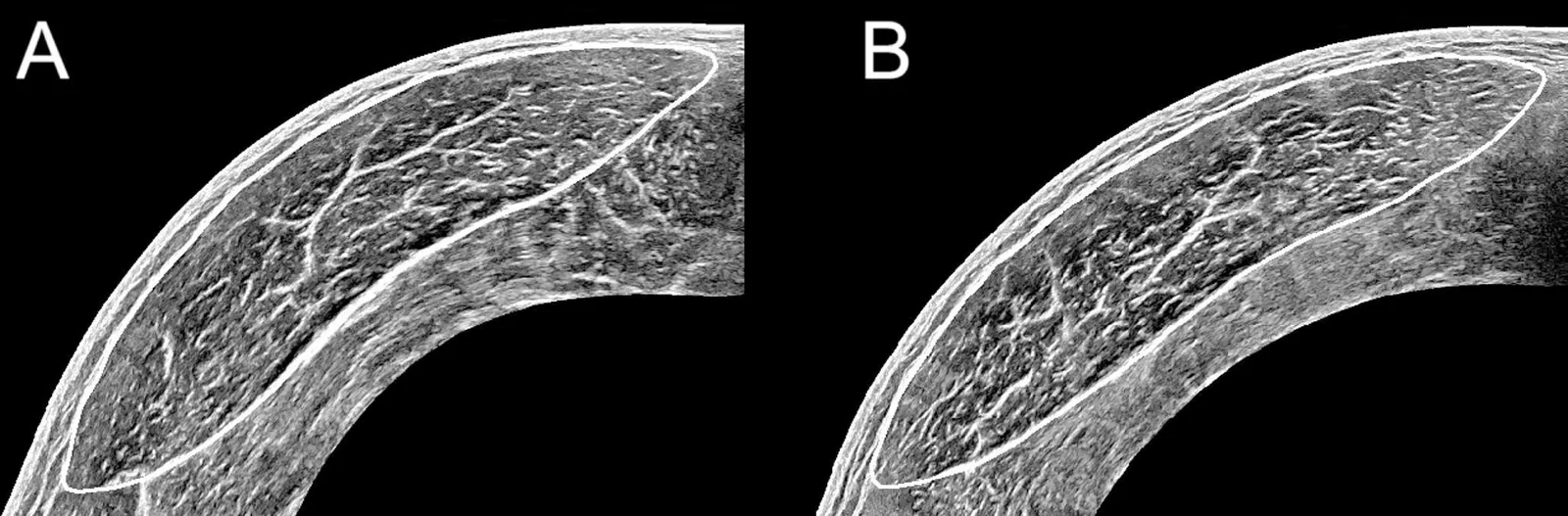
Representative images of the gastrocnemius medialis (GM) muscle anatomical cross-sectional area (ACSA) of an academy soccer player (**A**) and a control participant (**B**) measured at 50% GM length

Participants lay relaxed in the prone position on a therapy bed during the GM ultrasound scans, with their foot fixed at an ankle angle of 0° (neutral). A 40 mm wide ultrasound probe (18 − 5 MHz), operating in real-time B Mode (Philips Epiq 7, Guildford, UK), was placed on the skin in the sagittal plane to identify the distal and proximal MTJ of the GM, and these points were marked on the skin with an indelible pen. Muscle length was measured between these two points using an anthropometric tape measure (Seca 201, Liverpool, UK), and intervals at 20, 40, 50, 60 and 80% were marked along the muscle length. At each interval, the medial and lateral borders of the GM were located with the ultrasound probe in the transverse plane, which allowed for the midpoint at each section to be identified and marked on the skin. Thin (2 mm wide) strips of surgical tape (3 M Micropore, Bracknell, UK) were placed transversely at 50% muscle length to provide a visual reference point on the subsequent ultrasound scan performed in the sagittal plane. With the ultrasound probe placed sagittally and perpendicular to the skin, and water-soluble gel aiding transmission, an extended field of view (EFOV) image along the midline of the muscle was taken (Fig. 1), which was used to measure GM MT, *L*_f_, and *θ*_p_. With the ultrasound probe placed transversely at 50% muscle length, an EFOV image was taken from the medial to lateral border, which was used to measure GM ACSA (Fig. 2). In CON, GM ACSA was also measured at 20, 40, 60 and 80% muscle length, which enabled the calculation of GM *V*_m_ (see below). Minimal pressure was applied throughout the scans to avoid compression of soft tissue.

### Maximum voluntary contraction (MVC)

Participants were seated and firmly secured on an IKD (Humac Norm, CSMI, Massachusetts, USA), sat in an upright position (hip angle at 85°; supine position = 180°) with the knee of their dominant leg fully extended (0° knee joint angle) and ankle set in a neutral position (0°; 90° = full plantar flexion). The centre of rotation on the IKD lever arm was in line with the participants’ lateral malleolus. With the foot fixed in a foot plate attachment (secured by three inextensible straps), the thigh and torso were secured to the seat with inextensible straps to minimise additional movement or contribution to torque production from anything other than the plantar/dorsiflexor muscles. Participants completed a standardised warm up comprising 10 isokinetic contractions at 60°·s^− 1^, gradually increasing contraction intensity to near-maximal at repetition #10. Following the warm up, participants completed three PF and three DF isometric MVCs with the ankle set at neutral. Each trial alternated between plantar flexion and dorsiflexion, interspersed with 30 s rest and each MVC lasted for ~ 3 s until a plateau in the torque-time trace occurred.

### Countermovement jumps

Participants performed vertical and horizontal CMJs, both bilaterally and unilaterally. Vertical GRF (VGRF), horizontal anterior–posterior GRF (HGRF), and medio-lateral GRF (MGRF) data were collected using an in-ground 0.9 × 0.6 m^2^ force platform (9287 C, Kistler Instruments Ltd., Winterhur, Switzerland), at a sampling rate of 1000 Hz. Participants were instructed to stand still on the force platform for at least 3 s prior to initiation of the jump to provide a silent period in the force-time trace, thus allowing an accurate determination of countermovement onset (Owen et al. 2014). Regarding the unilateral CMJs [described in detail elsewhere (Murtagh et al. 2017; Robshaw et al. 2025)], an attempt was deemed acceptable if the femur of the contra-lateral limb did not pass behind the femur of the dominant limb in the sagittal plane of motion, as determined via visual inspection by the investigator. Participants were also required to land initially on their jumping leg before their non-jumping limb could come into contact with the floor to provide balance, which for the horizontal CMJs (HCMJs), had to be placed in front of the jumping limb. For the horizontal HCMJs, jump distance was recorded using a tape measure running parallel to the force platform and measured to the nearest 1.0 cm, and a dowel was placed against the heel of the jumping leg upon landing, perpendicular to the tape measure (Murtagh et al. 2017, 2018a, b; Robshaw et al. 2025). Jump height and projectile range were calculated for each vertical and horizontal CMJ, respectively (as outlined later), and the trial with the highest or furthest jump was used in subsequent analysis.

### Drop jumps

Details of the drop jump procedure have been published recently (Robshaw et al. 2025). Briefly, bilateral drop jumps were performed from a 40 cm box, where an attempt was only deemed acceptable if contact time was below 250 ms (Schmidtbleicher, 1992). Upon landing on the force platform, participants were cued to jump as high as possible, whilst minimising ground contact time. Reactive strength index (RSI) was calculated by dividing jump height by ground contact time, with jump height determined via flight time (Flanagan and Comyns 2008). Vertical stiffness, the amount of centre of mass displacement experienced in response to VGRF, was calculated by dividing peak vertical force by peak centre of mass displacement during ground contact (Farley and Morgenroth 1999; Maloney et al. 2016). For the drop jumps, the attempt with the highest RSI was used for subsequent analysis.

### Data processing

#### Muscle architecture

Three fascicles were identified at 50% muscle length in the sagittal scan (Fig. 1), all of which had to pass through the mid-point of the muscle (as identified by the ‘shadow’ produced by the micropore tape on the skin) and have a clear insertion into both the superficial and deep aponeuroses to prevent any need for extrapolation or estimation of length (Franchi et al. 2018). *L*_f_ was traced and measured from the insertion of deep to superficial aponeuroses using ImageJ software, while *θ*_p_ was measured from the same fascicles and taken from the angle of insertion into the deep aponeurosis. Muscle thickness was also determined at the mid-point of the muscle as the distance perpendicular to the superficial aponeurosis along the ‘shadow’ of the micropore tape. ASCA was identified using the transverse images (Fig. 2), by manually tracing the outline of the GM at the 50% muscle length interval, using ImageJ. Muscle volume (*V*_m_) was calculated in CON only by adopting a validated truncated cone method (Esformes et al. 2002), previously implemented for the GM (Erskine et al. 2017), and which utilised the ACSAs taken at 20, 40, 50, 60 and 80% muscle length intervals, using the equation, below:

$$V_{{\mathrm{m}}} = {\text{ 1}}/{\mathrm{3}} \cdot d \cdot \left[ {a + \surd \left( {a \cdot b} \right)\, + \,b} \right]$$

where *d* is the distance between ACSA intervals (*a* and *b*). GM muscle physiological cross-sectional area (PCSA) was then determined by dividing *V*_m_ by *L*_f_.

#### MVCs

For all plantar flexion and dorsiflexion MVCs, torque was recorded using AcqKnowledge software (Version 5, Biopac Systems Inc., Goleta, USA). For the analysis of peak torque, resting baseline torque values were subtracted from the peak torque recorded across trials with the highest peak torque taken as the MVC torque for both plantar flexion and dorsiflexion. Ankle moment and angle data were recorded via an analogue-to-digital converter (MP160, Biopac Systems Inc., Goleta, USA), sampled at 2000 Hz with a PC using AcqKnowledge software. Torque and angle data were filtered using a low-pass filter at a 10-Hz edge frequency.

### Jump data analysis

VGRF and HGRF were recorded throughout the jumping protocols in AcqKnowledge (Biopac). The raw force-time data were exported into a customised Excel Spreadsheet (Office 2007, Microsoft, Redmond, USA). Participant body weight (N), taken as an average from the ‘silent period’ prior to the onset of countermovement, was subtracted from VGRF to provide a value of net vertical force. Net horizontal force was taken from the force-time trace, as it was not considered to be influenced by gravity. Centre of mass acceleration was calculated for both vertical and horizontal directions, by dividing the respective net force by participant body mass (kg). Numerical integration of both vertical and horizontal acceleration provided a calculation of vertical and horizontal velocity, respectively, as per Newton’s laws of motion as described elsewhere (Blazevich 2007).

Jump height from the vertical CMJ trials was estimated using a calculation of constant acceleration (Dowling and Vamos 1993):

$$Take - off velocity^{2} /2*gravity$$

HCMJ distance was determined using a tape measure along with a formula for projectile range. Distance measured with a tape measure can be influenced by landing technique and limb length (Meylan et al. 2009), whereas projectile range is calculated using an equation incorporating the impulse-momentum relationship (Grimshaw et al. 2004):

$$\left( {Resultant Take - off Velocity^{2} * \left( {2*Sin Angle of Take - off} \right)} \right)/\left( {2*Gravity} \right)$$

Instantaneous power was measured by the sum of GRF vectors (vertical, anterior-posterior and medio-lateral) multiplied by their respective velocity for a given time point (Vigotsky et al. 2019):

$$P\, = \,F_{x} \cdot V_{x} + F_{y} \cdot V_{y} + F_{z} \cdot V_{z}$$

Peak power was taken as the highest power value calculated during stance.

For both countermovement protocols, the onset of countermovement was identified in line with current guidelines (Owen et al. 2014), as the first occurrence when the vertical force passed below five times the standard deviation of the silent period. Flight phase onset was determined as the time point when the corresponding vertical force passed below five times the standard deviation of the vertical force during flight phase. The attempt with the greatest jump height or projectile range was used for each participant, along with the associated variables for that attempt.

Jump height during drop jumps was calculated from an equation of flight time rather than take-off velocity, which can be influenced by the initial falling velocity (Moir 2008):

$$\left( {0.5 * 9.81} \right) * \left( {\left( {Flight Time/2} \right)^{2} } \right)$$

For each jump, flight time and contact time were determined using the same approach for identifying flight phase in the CMJs. Both variables were used in the calculation of RSI (Flanagan and Comyns 2008):

$$Flight time\left( s \right)/Contact time\left( s \right)$$

At the instance of ground contact, an initial velocity of -2.80 m/s^− 1^ was implemented, through an equation to determine instantaneous velocity of an object falling for a known height:

$$\surd \left( {2 * gravity * 0.4} \right)$$

Vertical velocity was integrated to calculate centre of mass displacement during the drop jumps and used in conjunction with vertical force to calculate vertical stiffness (Farley and Morgenroth 1999):

$$Peak vertical force \left( N \right)/ peak centre of mass displacement \left( m \right)$$

The jump with the highest RSI/jump height was used in further analysis.

### Statistical analysis

All variables were assessed to determine whether they met the parametric assumptions using a Levenes test for homogeneity of variance (*P* < 0.05) and a Shapiro-Wilk test to determine normal distribution (*P* < 0.05). If the data were normally distributed, differences between group means were determined using independent samples t-tests, however, if the data were not normally distributed (i.e. for unilateral vertical CMJ peak power, jump height and take-off velocity), a Mann Whitney-U independent test was implemented instead. Similarly, Pearson’s correlations were used to identify the relationship between muscle characteristics and jump performance for normally distributed data, whilst Spearman’s correlations were used for non-normally distributed data. A sub-sample of 10 CON participants attended the laboratory for a third time (2–7 days following the second visit) and were used to determine between-session reproducibility for all independent variables. Reproducibility was assessed using a two-way random effects model (absolute agreement) intraclass correlation coefficient (ICC), as well as ratio limits of agreement (Nevill and Atkinson 1997). Absolute reliability for all independent variables was reported as typical error (TE), while relative reliability was calculated using coefficient of variation (CV, %) (Hopkins 2000). TE was calculated using the following equation:

$$Standard Deviation of Differences / \left( {\surd 2} \right)$$

CV was determined using the following equation:

$$100 * \left( {\surd \left( {Sum of squared differences/\left( {2* sample size} \right)} \right)/\left( {Average of measurement 1 and 2} \right)} \right)$$

Unless otherwise stated, data are reported as mean and standard deviation (SD) for each independent variable on each testing occasion. All statistical analyses were completed using SPSS software (Version 30, IBM SPSS Inc., Chicago, USA), and statistical significance was accepted when *P* < 0.05.

## Results

### Reliability

Good to excellent between-session reliability for all morphological and architectural characteristics of the GM muscle was demonstrated by low CVs and TEs (Table 1). Furthermore, very strong ICC values with narrow 95% confidence intervals (*P* < 0.001) were found for ACSA, PCSA, *V*_m_, *L*_f,_ MT and *θ*_p,_ thus demonstrating high reproducibility for these variables. Small mean bias ratios were demonstrated for these parameters (1.00-1.03), which also indicates high test-retest reproducibility (Table 1). The close ratio limits of agreement (RLoA, */÷ 1.04–1.22) indicate that 95% of the agreement ratios for each variable lay within 4 to 22% above and below the respective mean bias ratios. For example, for GM ACSA, 95% of the agreement ratios lay within 4% above and below a mean bias ratio of 1.00, i.e. between a lower limit of agreement of 1.00 ÷ 1.04 = 0.96 and an upper limit of agreement of 1.00*1.04 = 1.04. Thus, it could be stated with 95% certainty that retest GM ACSA measurements were between 4% lower and 4% higher than the first measurements.

**Table 1:** Test-retest reproducibility of gastrocnemius medialis (GM) muscle morphological characteristics

Muscle characteristic	ICC	95% CI		*P* value	MBR (*/÷RLoA)	CV (%)	TE (units)
		Lower	Upper				
*L*_f_	0.933	0.772	0.981	< 0.001	1.01 (1.20)	6.46	0.39 (cm)
*θ*_p_	0.903	0.667	0.972	< 0.001	1.01 (1.22)	6.39	0.95 (°)
MT	0.985	0.949	0.996	< 0.001	1.01 (1.06)	2.20	0.04 (cm)
GM ACSA	0.998	0.993	0.999	< 0.001	1.00 (1.04)	1.19	0.17 (cm^2^)
GM *V*_m_	0.985	0.947	0.996	< 0.001	1.02 (1.10)	3.37	4.92 (cm^3^)
GM PCSA	0.919	0.713	0.978	< 0.001	1.03 (1.19)	6.19	1.45 (cm^2^)

*CI* confidence interval, *MBR* mean bias ratio, *RLoA* ratio limits of agreement, *L*_f_ fascicle length, *θ*_p_ fascicle pennation angle, *MT* muscle thickness, *ACSA* anatomical cross sectional area, *PCSA* physiological cross sectional area, *V*_m_ muscle volume, *ICC* intraclass correlation coefficient, *CV* coefficient of variation, *TE* typical error

Between-session reproducibility of all jump variables have been presented previously (Robshaw et al. 2025). In brief, coefficient of variation analysis for bilateral vertical CMJ variables revealed good to moderate values for all jump parameters (4.44–11.38%). Unilateral vertical CMJ test-retest reliability outputs displayed strong ICC values, with narrow 95% confidence intervals and low P values were found for take-off velocity, peak power and jump height, demonstrating a very good association between testing occasions. Slightly lower CVs were found for all parameters during unilateral vertical CMJs than for bilateral. Mean bias was similar across all variables and within a suitable range (0.97–1.05). Test-retest reliability of bilateral horizontal CMJ variables demonstrated strong values consistently for peak power, projectile range, resultant take-off velocity and measured distance. Reproducibility of unilateral horizontal CMJ variables followed a similar pattern to the bilateral equivalent outlined above. Inter-session reproducibility values for jump height, flight time and contact time during vertical drop jumps produced the lowest CVs amongst all variables (5.35–9.91%), whilst reactive strength index, vertical stiffness and peak force demonstrated higher CVs (10.2–21.5%).

### Between group differences in gastrocnemius medialis muscle properties

**Fig. 3:**
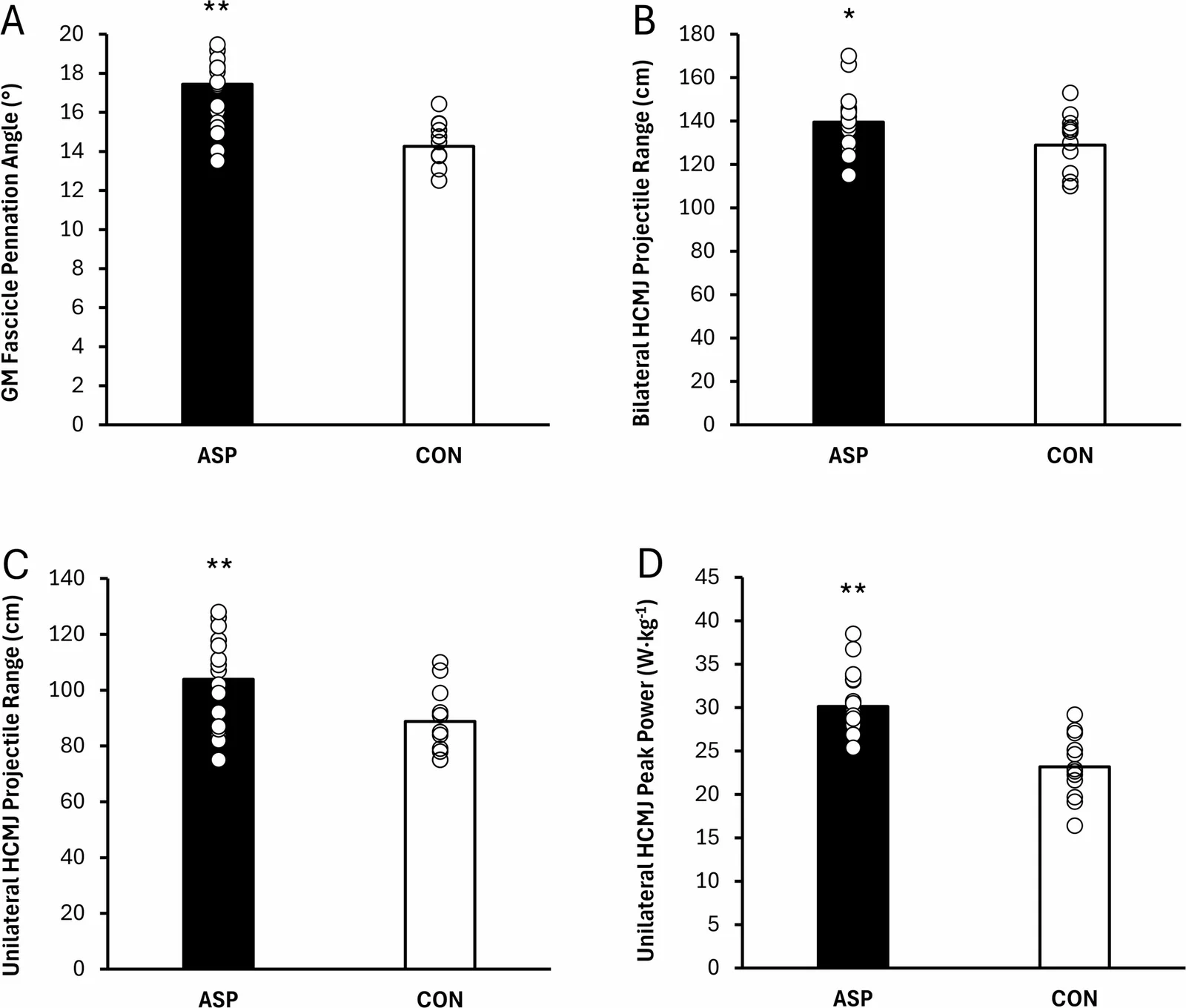
Differences between academy soccer players (ASP) and control participants (CON) in (**A**) gastrocnemius medialis (GM) fascicle pennation angle (*θ*_p_); (**B**) projectile range during a bilateral horizontal countermovement jump (HCMJ); (**C**) projectile range during a unilateral HCMJ; and (**D**) peak power during a unilateral HCMJ. * *P* < 0.05, ** *P* < 0.01 different to CON

Morphological characteristics of the GM muscle for ASP and CON are displayed in Table 2. Muscle *θ*_p_ was larger in ASP compared to CON (*P* < 0.001, Cohen’s *d* = 1.482, Fig. 3) but no other differences were found between groups. Peak PF torque (209 ± 35 vs. 206 ± 38 Nm, *P* = 0.804) and peak DF torque (37.0 ± 6.0 vs. 34.1 ± 5.4 Nm, *P* = 0.180) did not differ between ASP and CON.

**Table 2:** Morphological characteristics of the gastrocnemius medialis (GM) muscle in academy soccer players (ASP) and recreationally active control participants (CON). Values are mean (SD)

Variable	ASP	CON	*P* value
ACSA (cm^2^)	14.30 (2.47)	13.90 (2.58)	0.296
*L*_f_ (cm)	5.91 (0.93)	6.24 (1.04)	0.352
*θ*_p_ (°)	17.40 (2.49)*	14.30 (1.23)	0.0002
MT (cm)	1.83 (0.21)	1.81 (0.24)	0.516

*CSA* cross sectional area, *L*_f_ fascicle length, *θ*_p_ fascicle pennation angle, *MT* muscle thickness, * different to CON

### Between group differences in jump performance

Table 3 displays the between group differences for primary jump variables. Between group differences were found for peak power in unilateral horizontal CMJ (*P* < 0.001, Cohen’s *d* = 1.934, Fig. 3). Projectile range was also greater in ASP for both bilateral (*P* = 0.041, Cohen’s *d* = 0.772, Fig. 3) and unilateral (*P* = 0.006, Cohen’s *d* = 1.056, Fig. 3) horizontal CMJs, whilst no between group differences were found for either the vertical CMJ or drop jump assessments.

**Table 3:** Jump variables for academy soccer players and recreationally active control participants. Values are mean (SD)

Jump type	Variable (units)	ASP	CON
Bilateral VCMJ	Jump height (cm)	43.9 (6.0)	40.7 (5.0)
	Peak power (W kg^− 1^)	30.6 (4.1)	32.0 (4.6)
Unilateral VCMJ	Jump height (cm)	24.5 (2.9)	22.7 (7.1)
	Peak power (W kg^− 1^)	17.9 (4.3)	16.7 (5.6)
Bilateral HCMJ	Projectile range (cm)	140 (14) *	129 (14)
	Peak power (W kg^− 1^)	39.6 (5.1)	37.18 (4.9)
Unilateral HCMJ	Projectile range (cm)	104 (16)**	89 (12)
	Peak power (W kg^− 1^)	30.14 (3.53)**	23.18 (3.72)
Bilateral Drop Jump	Contact time (s) Flight time (s) Jump height (cm) Reactive strength index (AU) Vertical stiffness (Nm·kg^− 1^)	0.197 (0.017) 0.520 (0.047) 33.3 (5.9) 2.65 (0.34) 478 (197)	0.206 (0.017) 0.507 (0.026) 31.3 (3.4) 2.48 (0.24) 437 (218)

*VCMJ* vertical countermovement jump, *HCMJ* horizontal countermovement jump

*P < 0.05, **P < 0.01 different to CON

### Relationships between gastrocnemius medialis muscle properties and jump performance

**Fig. 4:**
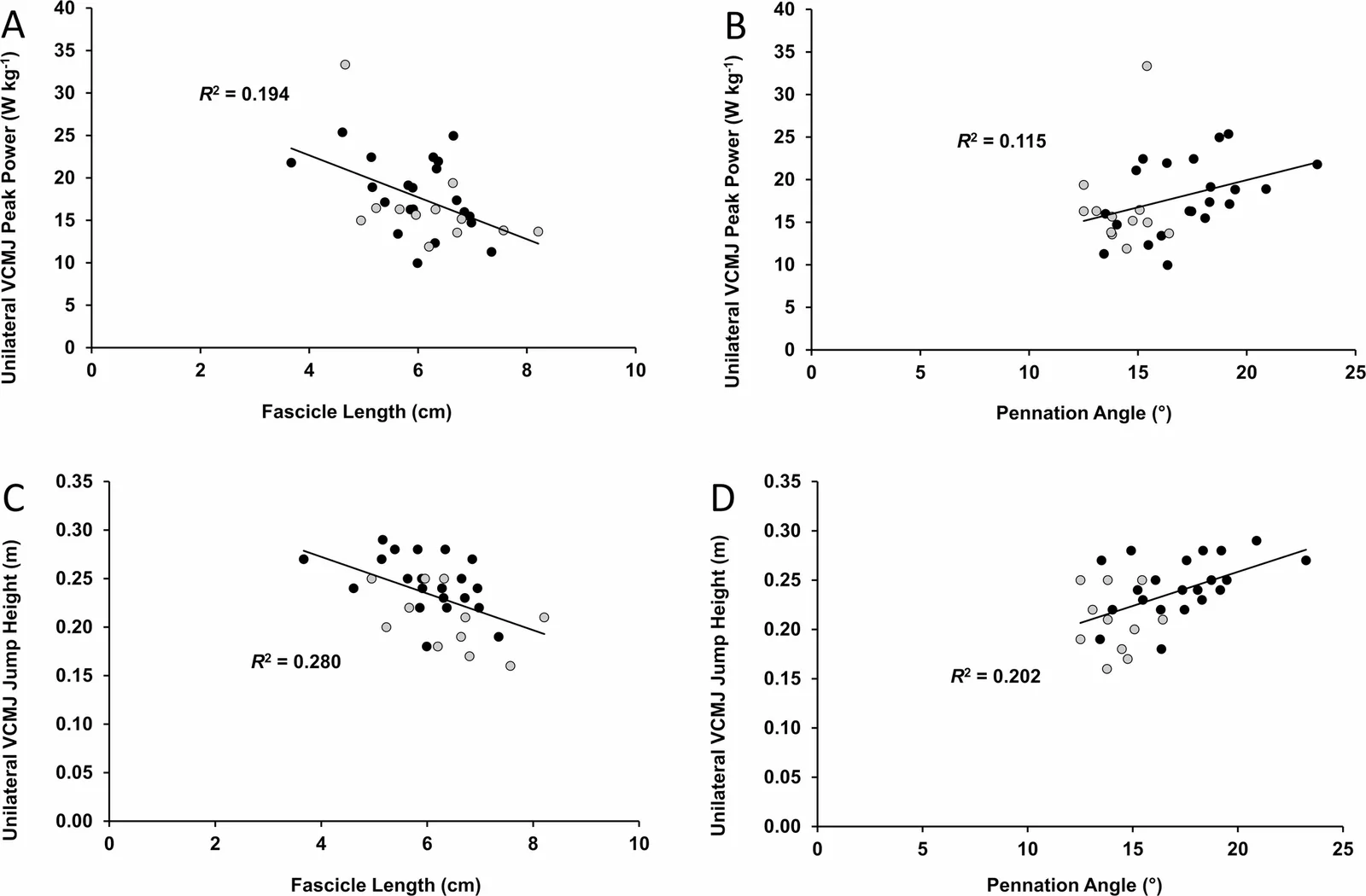
Relationship between unilateral vertical countermovement jump (CMJ) peak power and **A** GM *L*_f_ (*r* = -0.441, *P* = 0.01) and **B** *θ*_p_ (*r* = 0.339, *P* = 0.021) in ASP (black data points) and CON (grey data points). Relationship between unilateral vertical countermovement jump (CMJ) jump height and **C** GM *L*_f_ (*r* = -0.529, *P* = 0.002) and **D** *θ*_p_ (*r* = 0.449, *P* = 0.009) in ASP (black data points) and CON (grey data points)

**Fig. 5:**
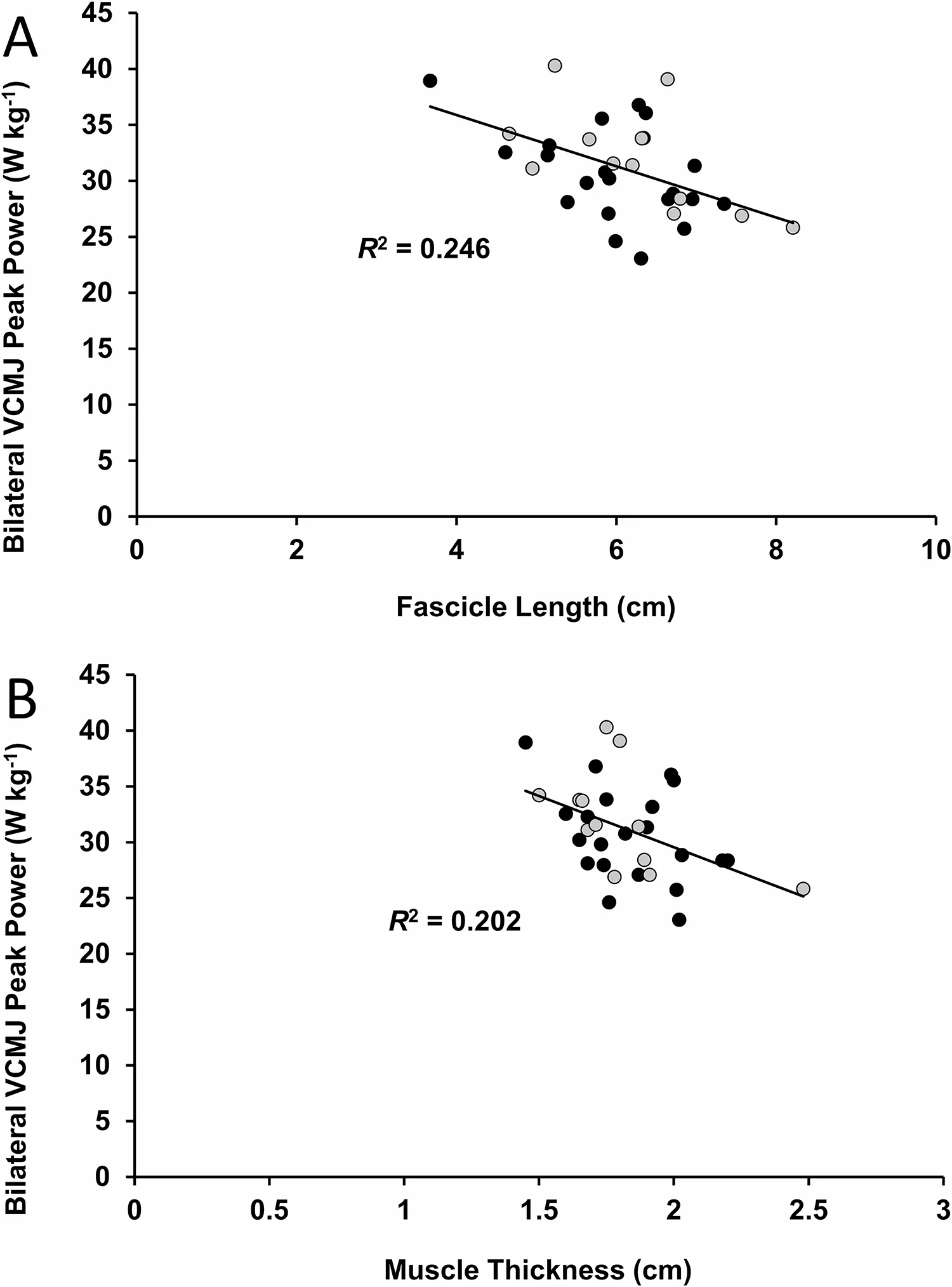
Relationship between bilateral vertical countermovement jump (CMJ) and **A** GM *L*_f_ (*r* = − 0.496, *P* = 0.003), and **B** GM muscle thickness (*r* = − 0.449, *P* = 0.009) in ASP (black data points) and CON (grey data points)

Correlations (both groups combined) were found between morphological characteristics and jump variables (Figs. 4 and 5). GM *L*_f_ correlated *inversely* with unilateral VCMJ height (*r* = − 0.529, *P* = 0.002, Fig. 4), unilateral VCMJ peak power (*r* = − 0.441, *P* = 0.010, Fig. 4) and peak power during bilateral VCMJ (*r* = − 0.496, *P* = 0.003, Fig. 5). GM *θ*_p_ correlated *positively* with unilateral VCMJ height (*r* = 0.449, *P* = 0.009, Fig. 4) and peak power (*r* = 0.339, *P* = 0.021, Fig. 4), while GM MT correlated *inversely* with peak power during a bilateral VCMJ (*r* = − 0.449, *P* = 0.009, Fig. 5). No other significant correlations between GM morphological characteristics and jump variables were found when both groups were combined, although peak PF torque correlated *positively* with bilateral drop jump peak force (*r* = 0.482, *P* = 0.005).

Within ASP alone, GM *L*_f_ correlated *inversely* with unilateral VCMJ height (*r* = − 0.447, *P* = 0.042) but *positively* with peak power during bilateral HCMJ (*r* = 0.452, *P* = 0.040). Conversely, GM *θ*_p_ correlated *positively* with unilateral VCMJ height (*r* = 0.440, *P* = 0.046) and unilateral VCMJ take-off velocity (*r* = 0.443, *P* = 0.044), but *inversely* with unilateral HCMJ projectile range (*r* = − 0.468, *P* = 0.032) and take-off velocity (*r* = -0.464, *P* = 0.034).

In CON only, GM ACSA correlated *inversely* with unilateral (*r* = − 0.797, *P* = 0.002) and bilateral (*r* = − 0.741, *P* = 0.006) VCMJ peak power. GM MT correlated *inversely* with unilateral (*r* = − 0.669, *P* = 0.017) and bilateral (*r* = − 0.755, *P* = 0.005) VCMJ take-off velocity; with unilateral (*r* = − 0.674, *P* = 0.016) and bilateral (*r* = − 0.684, *P* = 0.014) VCMJ jump height; and with unilateral (*r* = − 0.657, *P* = 0.020) and bilateral (*r* = − 0.608, *P* = 0.036) VCMJ peak power. GM *V*_m_ correlated *inversely* with bilateral VCMJ height (*r* = − 0.791, *P* = 0.006) and take-off velocity (*r* = − 0.855, *P* = 0.002). GM *L*_f_ correlated *inversely* with unilateral (*r* = − 0.613, *P* = 0.034) and bilateral (*r* = − 0.762, *P* = 0.004) VCMJ height; with unilateral (*r* = − 0.619, *P* = 0.032) and bilateral (*r* = − 0.781, *P* = 0.003) VCMJ take-off velocity; and with bilateral VCMJ peak power (*r* = − 0.605, *P* = 0.037).

## Discussion

The aims of this study were to investigate (i) the reproducibility of GM muscle characteristics; (ii) plantar flexion strength and GM muscle morphology in a group of ASP and CON participants; and (iii) the relationship between these characteristics and jump performance. The main findings were that (i) GM muscle characteristics were highly reproducible; (ii) ASP had a larger GM *θ*_p_ than CON (although no other differences existed between groups regarding GM muscle architecture), and were capable of producing greater projectile range with higher power outputs during horizontal (but not vertical) CMJs (there were no differences in drop jump performance between ASP and CON); and (iii) that GM muscle architecture displayed direction-specific correlations with CMJ performance in ASP, while CON displayed only inverse correlations with CMJ performance, and only in the vertical plane. This suggests GM muscle architecture plays an important role in CMJ performance in ASP, and that the contribution of this muscle’s characteristics to CMJ performance differs between ASP and CON.

We found that GM *θ*_p_ was greater in ASP than CON (Fig. 3), with no other between group differences in GM muscle morphology or architecture. A larger *θ*_p_ is thought to represent a greater number of sarcomeres arranged in parallel, which will result in a larger muscle fibre cross-sectional area (Aagaard et al. 2001; Degens et al. 2009; Gollnick et al. 1981). In turn, this provides a larger quantity of contractile tissue attaching to the aponeurosis, which provides a greater capacity to generate force (Aagaard et al. 2001; Degens et al. 2009; Kawakami et al. 1993). Differences in *θ*_p_ could therefore represent innate characteristics possessed by ASP or a training adaptation. As our ASP were not completing any plantar flexor-specific resistance training at the time of performing this cross-sectional study, it is possible that any adaptation could have been induced by the habitually high GRF and muscular forces generated during pitch-based academy soccer training, whereby consistent exposure to activities requiring large force production may result in architectural adaptation (Lai et al. 2016; Verheul et al. 2024). However, this hypothesis remains speculative and investigation into the neuromuscular adaptations following habitual soccer-specific training and match-play is warranted.

Despite limited variation in GM architecture between our ASP and CON groups, several differences existed in the jump assessments (Table 3; Fig. 3). Peak power during a unilateral horizontal CMJ was greater in ASP, whilst no differences in power were found during either the bilateral horizontal or vertical CMJs. These findings are in support of previous investigations, which suggest utilising unilateral jumps as a more informative assessment over bilateral jumps to differentiate between ASP and CON (Meylan et al. 2009; Murtagh et al. 2017), potentially a result of the higher forces required in unilateral CMJ (Bishop et al. 2022). Similarly, projectile range during a unilateral horizontal CMJ was greater in our ASP compared to CON, a finding which replicates that of a previous independent study by Murtagh et al. (2017). Future research should therefore use projectile range and power from unilateral horizontal CMJs to compare between different athletic populations, and to track time-course adaptations to training interventions. In contrast, none of the performance variables from a drop jump differed between ASP and CON. A lack of difference might be explained by the relatively poor reliability of drop jump outputs (Robshaw et al. 2025), where vertical stiffness and RSI had a between-session CV of 18.7% and 10.2%, respectively. However, despite the high reproducibility of all GM muscle size and architecture variables measured in this study (Table 1), which was similar to previously reported reliability values for GM architecture (Mohagheghi et al. 2007; Ritsche et al. 2022; McMahon et al. 2016; Raj et al. 2012), only *θ*_p_ differed between groups. This suggests low reproducibility may not be the only explanation for no between group differences in drop jump performance. An alternative reason could be related to powerful actions being performed less often in the vertical compared to horizontal plane during U18 and U21 ASP match-play (Murtagh et al. 2019), and may therefore not be prioritised in ASP training.

Whilst investigating potential differences in GM morphology and jumping ability between a group of ASP and CON was the primary aim of the present study, a second aim was to correlate these variables to better understand the physiological determinants of jump performance. When both ASP and CON were combined, GM muscle architecture correlated with jump performance in a specific manner (e.g. *L*_f_ correlated inversely with vertical jump performance, while *θ*_p_ correlated positively). The coefficients of determination (*R*^2^ = 0.12–0.28, Figs. 4 and 5) suggest GM architecture explains ~ 12–28% of the variability in CMJ performance, which is in line with previous reports for the GL (Earp et al. 2010) but lower than for larger muscles, such as *m. vastus lateralis* (Secomb et al. 2015a, b; Murtagh et al. 2018a, b a). The relatively low but significant coefficients of determination for the GM are unsurprising, considering the GM is just one muscle contributing to CMJ performance, which is a complex, multi-joint task, involving numerous lower-limb muscles.

Our direction-specific correlations between GM muscle architecture and CMJ performance were further emphasised when analysis was limited to ASP only. For example, GM *L*_f_ demonstrated *inverse* correlations with jump performance in the vertical plane and *positive* correlations in the horizontal plane. The latter finding may be linked to *L*_f_ being the main determinant of a muscle’s maximum shortening velocity (Degens et al. 2009; Jones et al. 1989), as well as the greater contribution of the ankle to propulsion during horizontal than vertical jumps (Kotsifaki et al. 2021). In contrast, the inverse relationship between *L*_f_ and *vertical* CMJ performance might be explained by muscle-tendon interactions during a CMJ (Kurokawa et al. 2001, 2003). Changes to the muscle-tendon unit length during a CMJ are primarily explained by lengthening and shortening of the tendinous tissue whilst muscle fibres remain close to their optimal length, contracting quasi-isometrically or shortening at slow contractile velocities (Farris et al. 2016; Kurokawa et al. 2001, 2003).

Furthermore, the GM appears to favour operating over the ascending limb of the length-tension relationship during running and sprinting (Lai et al. 2014; Monte et al. 2020). Previous studies have reported a progressive shift of fascicles to shorter, less favourable operating lengths with increasing running speeds, which also undergo less overall length change (Lai et al. 2014; Monte et al. 2020; Schwaner et al. 2024). A shorter fascicle may enable the individual sarcomeres to remain closer to their optimum length for force generation, whereas a longer fascicle would require greater individual sarcomere shortening to reach the same overall length of fascicle. This could potentially reduce the number of cross-bridges available to generate force. Therefore, shorter fascicles that are more densely packed in parallel (through an increase in *θ*_p_) may be inducive to optimal GM muscle-tendon unit behaviour during vertical jumping tasks, enabling a greater force production, which can increase the elastic energy stored in the attaching tendon and, in turn, increase power output.

In direct contrast to the *L*_f_ correlations in ASP, GM *θ*_p_ (which was larger in ASP than CON) displayed *positive* correlations with CMJs in the vertical plane and *inverse* correlations in the horizontal plane. The positive correlations in the vertical plane may be linked to the number of sarcomeres arranged in parallel [i.e. the main determinant of muscle force (Degens et al. 2009; Jones et al. 1989)]. However, a large *θ*_p_ can limit whole muscle contraction velocity, thus limiting power during horizontal propulsion (Degens et al. 2009) and potentially explaining the inverse correlations in the horizontal plane.

The diminutive size of the GM (e.g. compared to larger muscles, such as the quadriceps) may explain our lack of any relationship between GM muscle size and jump performance in ASP. However, in CON only, we found inverse correlations between GM muscle size characteristics (e.g. anatomical cross-sectional area, ACSA; muscle thickness, MT; and muscle volume, *V*_m_) and CMJ performance in the *vertical* plane only. Although *V*_m_ was only measured in CON, the contrasting correlation patterns between ASP and CON suggest ASP GM muscle architectural characteristics play a different role in CMJ performance compared to CON. Indeed, these findings in CON suggest a smaller GM is beneficial for generating peak power, which may seem counterintuitive considering power is the product of force [primarily determined by muscle PCSA (Powell et al. 1984)] and velocity [principally influenced by muscle *L*_f_ (Lieber and Ward 2011)], and *V*_m_ is the product of PCSA × *L*_f_ (Erskine et al. 2011). However, in individuals who do not habitually perform jumping actions pertinent to soccer (e.g. CON), lower neuromuscular activation capacity may restrict the ability to utilise larger muscles to generate peak power. Additionally, while larger muscles can generate higher absolute force (Erskine et al. 2014), a concomitant greater body mass can outweigh the gain in peak power (Javiland et al. 2018). This may be exacerbated during vertical CMJs, where the mechanical constraint of gravity opposing motion is greater than the load represented by body weight (Samozino et al. 2013). Evidently, future studies are required to elucidate the physiological and biomechanical bases for these unexpected relationships between GM muscle size and vertical CMJ performance in CON.

We acknowledge this study has several limitations. Firstly, the ASP group consisted of U23 and U18 male soccer players recruited from a single English Premier League Category One soccer academy. Although this increases internal validity, it may limit the generalisability of findings to male players of similar ages in other academy settings, as well as to female players. Thus, future studies should aim to replicate these findings across different cohorts, including female academy soccer players, to explore whether sex-related differences exist in the GM characteristics that influence jump performance. Secondly, CON included age-matched males participating in a range of sports, not exclusively soccer. Although this prevents a direct comparison between ‘elite’ and ‘non-elite’ soccer players, it does provide a broader reference point by situating the ASP group within a sample of physically active, healthy young adult males.

To conclude, our study found that ASP displayed a larger GM *θ*_p_, and greater unilateral horizontal CMJ peak power and projectile range, compared to CON. The opposing *θ*_p_ and *L*_f_ correlations with vertical and horizontal CMJ jump performance in ASP suggest GM architecture influences CMJ performance in a direction-specific manner in this population. Furthermore, the different pattern of correlations between ASP and CON suggests that GM architecture contributes to CMJ performance differently between these two populations. Whether these differences in GM *θ*_p_ and CMJ performance are due to superior innate GM characteristics in ASP, or specific adaptation to habitual soccer training and match-play is unknown and requires further investigation.

## Data Availability

All data discussed in this article are presented in the tables/figures.
